# Crosstalk between gut microbiota and tumor: tumors could cause gut dysbiosis and metabolic imbalance

**DOI:** 10.1002/1878-0261.13763

**Published:** 2024-11-26

**Authors:** Siyuan Zhang, Haimei Wen, Ying Chen, Jingya Ning, Di Hu, Yujiao Dong, Chenyu Yao, Bo Yuan, Shuanying Yang

**Affiliations:** ^1^ Department of Respiratory and Critical Care Medicine The Second Affiliated Hospital of Xi'an Jiaotong University China; ^2^ School of Medicine Xi'an Jiaotong University China; ^3^ Department of Dermatology The Second Affiliated Hospital of Xi'an Jiaotong University China; ^4^ Xi'an Jiaotong University School of Medicine Affiliated Honghui Hospital China; ^5^ Department of Pathology The First Affiliated Hospital of Xi'an Jiaotong University China

**Keywords:** fecal microbiota transplantation, gut microbiota, metabolism, metagenomic sequencing, metastatic tumor, subcutaneous tumor

## Abstract

Gut microbiota has a proven link with the development and treatment of cancer. However, the causality between gut microbiota and cancer development is still unknown and deserves exploration. In this study, we aimed to explore the alterations in gut microbiota in murine tumor models and the crosstalk between the tumor and the gut microbiota. The subcutaneous and intravenous murine tumor models using both the colorectal cancer cell line MC38 and lung cancer cell line LLC were constructed. Then fecal samples before and after tumor inoculation were collected for whole metagenomics sequencing. Both subcutaneous and metastatic tumors markedly elevated the α‐diversity of the gut microbiota. Relative abundance of *Ligilactobacillus* and *Lactobacillus* was reduced after subcutaneously inoculating tumor cells, whereas *Bacteroides* and *Duncaniella* were reduced in metastatic tumors, regardless of tumor type. At the species level, *Lachnospiraceae bacterium* was enriched after both subcutaneous and intravenous tumors inoculation, whereas levels of *Muribaculaceae bacterium Isolate‐110 (HZI)*, *Ligilactobacillus murinus* and *Bacteroides acidifaciens* reduced. Metabolic function analysis showed that the reductive pentose phosphate cycle, urea cycle, ketone body biosynthesis, ectoine biosynthesis, C4‐dicarboxylic acid cycle, isoleucine biosynthesis, inosine 5′‐monophosphate (IMP), and uridine 5′‐monophosphate (UMP) biosynthesis were elevated after tumor inoculation, whereas the cofactor and vitamin biosynthesis were deficient. Principal coordinates analysis (PCoA) showed that subcutaneous and metastatic tumors partially shared the same effect patterns on gut microbiota. Furthermore, fecal microbiota transplantation revealed that this altered microbiota could influence tumor growth. Taken together, this study demonstrated that both colorectal cancer (MC38) and non‐colorectal cancer (LLC) can cause gut dysbiosis and metabolic imbalance, regardless of tumor type and process of tumor inoculation, and this dysbiosis influenced the tumor growth. This research gives novel insights into the crosstalk between tumors and the gut microbiota.

## Graphical abstract

In this research, we analyzed the relationship between gut microbiota and tumor. We discovered that both subcutaneous and metastatic tumors would alter the composition and metabolic function of gut microbiota. Meanwhile, fecal microbiota transplantation also indicated the anti‐tumor role of the gut microbiota, revealing the crosstalk between tumor and gut microbiota.

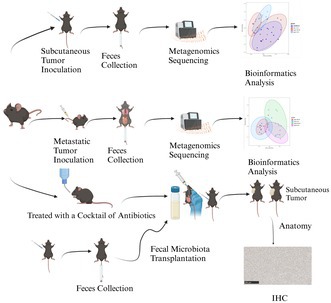

**Abbreviations**
BSA: Bovine serum album
DMEM: Dulecco's modified Eagle medium
F/B: firmicutes/bacteroidetes
FBS: fetal bovine serum
FMT: fecal microbiota transplantation
HE: hematoxylin–eosin
IHC: immunohistochemistry
IMP: inosine 5′‐monophosphate
KEGG: Kyoto Encyclopedia of Genes and Genomes
LDA: linear discriminant analysis
LEfSe: linear discriminant analysis effect size
LLC: Lewis lung cancer cell
OTU: operational taxonomic unit
PBS: phosphate buffered saline
PCoA: principal co‐ordinates analysis
SPF: specific pathogen free
STR: short tandem repeat
UMP: uridine 5′‐monophosphate
ZO‐1: Zona occludens 1

## 1 Introduction

Nowadays, it has been shown that the development of cancer is the result of multifactorial factors: genetics, age, environment, geographic location, diet, medications, infections, and chronic inflammation are all risk factors for the development of cancer [1]. Among them, diet is closely linked to gut microbiota. The gut microbiota is an ever‐changing ecosystem containing trillions of bacteria that can be constantly shaped by factors such as diet [2]. In recent years, numerous studies have demonstrated a strong relationship between gut microbiome disorders and various noncommunicable diseases, including cancer [3].

The relationship between gut microbiota and cancer has received increasing scholarly attention over the past few years. However, most studies have focused on the relationship between gut microbiome and gastrointestinal tumors, with little concerning about the relationship between gut microbiome and non‐gastrointestinal tumors [4]. Although the gastrointestinal and respiratory tracts are physically distant, they share the same embryonic origin and they have a high degree of structural similarity [5]. A published review concludes the presence of the gut–lung axis between gut microbiota and lung cancer [6]. The gut–prostate axis is another emerging axis. It has been found that high risk prostate cancer patients have a specific gut microbial profile and modification of the gut microbiota with antibiotics stimulates the development of prostate cancer. Further research on the “gut‐prostate axis” will help to identify new strategies for the prevention, screening, and treatment of prostate cancer [7]. Therefore, further exploration of its oncogenic mechanism and search for new tumor biomarkers can be helpful in the early diagnosis and treatment of cancer.

However, the causal relationship between tumors and gut microbiota still remains elusive. It has been found that gut microbiota profiles changed at different cancer stages, and some specific species are correlated with tumor stage [8]. Long et al. [9] showed several different taxa microbes were causal related with cancer using Mendelian randomization analysis. Yonekura also came to a similar conclusion that cancer could cause stress ileopathy and induce gut dysbiosis [10]. However, previous studies failed to specify the influence of different cancer types and stages on gut microbiota, as well as whether this altered microbiota could influence the tumor growth.

In this study, we hypothesized that the composition of the intestinal fecal changes before and after tumor inoculation, and the microbiota, as a protective factor, would inhibit tumor growth to some extent. To verify our hypothesis, the MC38 cell line, representing the colorectal cancer, as well as LLC cell line, representing non‐colorectal tumor, were utilized to establish subcutaneous and metastatic tumor model, and the whole metagenomics sequences were conducted to analyze the feces collected before and after tumor inoculation. The structure of colorectums before and after tumor inoculation was also observed. Moreover, we assayed the influence of these changed microbiota on tumor growth based on fecal microbiota transplantation (FMT). Our findings will give novel evidence on the causality of tumors and gut microbiota.

## 2 Materials and methods

### 2.1 Animal models

The 5–6 week old C57 BL/6J male mice (SPF grade) used in this study were purchased from Laboratory Animal Center of Xi'an Jiaotong University. All animal experiments were approved by the Animal Care and Research Ethics Committee of the Laboratory Animal Center of Xi'an Jiaotong University, China (Approval number: XJTUAE2023‐1206). The mouse experiments were conducted according to the GB/T 42011‐2022 and GB/T 35892‐2018 (the Chinese national guidelines for laboratory animal ethics).

All mice were fed with an ordinary diet and sterile water under SPF conditions in the Laboratory Animal Center of Xi'an Jiaotong University. The subcutaneous tumor model was inoculated with murine colon cancer cell line MC‐38 (3 × 10^5^/site) and Lewis lung cancer cell (LLC) line (5 × 10^5^/site), respectively, in the right axilla of mice. Metastatic tumor model was constructed by intravenous injection of MC‐38 cell line (3 × 10^5^/mouse) or LLC cell line (5 × 10^5^/mouse). Tumor volume was measured with the following formula: volume = (length × width^2^)/2. When tumor reached appropriate volume, the mice were sacrificed with 1.25% 2,2,2‐Tribromoethanol (T903147, Macklin, Shanghai, China; 0.2 mL/10 g weight), and tumor, colorectum, and lung tissues were resected carefully. Collected fecal samples were quick‐frozen in liquid nitrogen for 15 min and then stored at −80 °C until used.

For FMT, fecal samples were collected from donor mice before (marked as pre feces) and after (marked as post‐MC38 and post‐LLC feces) MC38 or LLC cell inoculation, respectively. Every 1 g fecal samples were resuspended in 10 mL sterile phosphate‐buffered saline (PBS) and the fecal suspension was centrifuged to remove visibly large particles and insoluble materials. Recipient mice involved in FMT were treated with a cocktail of antibiotics for 7 days first. The broad‐spectrum antibiotics contain ampicillin (A6265, Macklin; 1 g·L^−1^), colistin (C805491, Macklin; 1 g·L^−1^), neomycin (N6063, Macklin; 5 g·L^−1^), and vancomycin (V6062, Macklin; 0.5 g·L^−1^) diluting in sterile drinking water. This antibiotics cocktail containing β‐lactam (ampicillin), glycopeptide (vancomycin), aminoglycoside (neomycin), and polypeptide antibiotic (colistin), which has been proved effectively to eliminate gut microbes, including both gram‐positive and negative strains [4, 11, 12]. Then, 2 weeks prior to the tumor inoculation, the fecal suspension or sterile PBS was injected orally at a dose of 200 μL·mouse^−1^·day^−1^ with the gavage needle until the resection of tumor.

### 2.2 Metagenomics sequencing and 16S rRNA sequencing for fecal samples and data analysis

The detailed procedure was described previously [13]. Briefly, total genomic DNA was extracted from mice fecal samples using E.Z.N.A.Soil DNA Kit (M5635‐02; Omega Bio‐tek Inc., Norcross, GA, USA) following the manufacturer's instructions. Sequencing libraries were generated using Hieff NGS® MaxUp II DNA Library Prep Kit for Illumina® (12200ES96, YEASEN, Shanghai, China) following manufacturer's instructions, and index codes were added to attribute sequences to each sample. In a nutshell, DNA was broken into fragments of about 500 bp using Covaris 220. The library fragments were purified with Hieff NGS™ DNA Selection Beads DNA (12601ES56, YEASEN). The purified DNA was end repaired, adapter ligated, and fragment selection. fastp (version 0.36) [14] was used for evaluating the quality of sequenced data. After the raw reads were filtered, the remaining clean data was used for further analysis.

The resulting relative abundance profiles for all samples were imported into R for subsequent analysis. β‐diversity evaluated differences in the microbiome among samples. These analyses were visualized using r vegan package (version 2.5‐6), and finally, the inter‐sample distances were presented as scatterplots. The differential microorganisms between multiple groups were analyzed by lefse (version 1.1.0) [15]. Venn diagram was generated using the funrich software (version 3.1.3) [16]. diamond (version 0.8.20) [17] was used to compare the gene set with Kyoto Encyclopedia of Genes and Genomes (KEGG) database to obtain functional annotation information of genes. Based on gene set abundance information, functional abundance was obtained, and differential analysis between groups were analyzed using microbiotaprocess package (version 1.14.0) [18] in r. The α‐diversity was evaluated using the ACE, Shannon, and Chao1 diversity indices.

For 16S rRNA sequencing, V3–V4 hypervariable region of the 16S rDNA were amplified using 341F primer (CCTACGGGNGGCWGCAG) and 805R primer (GACTACHVGGGTATCTAATCC). Sequencing was performed using the Illumina MiSeq system (Illumina, San Diego, CA, USA), according to the manufacturer's instructions. After sequencing, the two short Illumina readings were assembled by pear software (version 0.9.8) [19] and the effective tags were clustered into operational taxonomic units (OTUs) of ≥ 97% similarity using _Usearch_ software (version 11.0.667) [20]. Representative sequences were classified taxonomically by blasting against the Silva Database. The α‐diversity and β‐diversity were evaluated as previously described.

### 2.3 Cell culture

Murine lung cancer cell line LL/2 (LLC1) (RRID: CVCL_4358) and murine colon cancer cell line MC‐38 (RRID: CVCL_B288) were obtained from iCell (Shanghai, China). LLC cell line was cultured with Dulecco's Modified Eagle Medium (DMEM; VivaCell, Shanghai, China) containing 10% fetal bovine serum (FBS; VivaCell) at 37 °C under 5% of CO_2_. MC38 cell line was cultured with DMEM containing 10% FBS and 1% HEPES (Procell, Wuhan, China) at 37 °C under 5% CO_2_. All experiments were performed with mycoplasma‐free cells. All cell lines were authenticated using short tandem repeat (STR) analysis as described in 2012 in ANSI Standard (ASN‐0002) by the ATCC Standards Development Organization (SDO) in the past 3 years.

### 2.4 Immunohistochemistry and HE staining

Colorectum, tumor, and lung tissues were carefully harvested and then fixed with 4% paraformaldehyde. Colorectum Swiss rolls were performed before fixed. Then the fixed tissues were dehydrated, paraffin embedded, and cut into 4‐μm thick paraffin sections. Subsequently, the sections were roasted, dewaxed, and hydrated.

For immunohistochemistry (IHC), the tissue slices were boiled in pressure cooker immersed in citrate buffer for 30 min to retrieve antigen followed by blocking with 3% hydrogen dioxide for another 20 min. Then, the slices were blocked with 5% bovine serum album (BSA) for 1 h at room temperature. The primary antibody used in this study include Ki‐67 (28074‐1‐AP, Proteintech, Wuhan, China; 1 : 1000) and ZO‐1 (21773‐1‐AP, Proteintech; 1 : 3000). The slices were incubated with primary antibody at 4 °C overnight and subsequently incubated with secondary antibody (MXB Biotechnologies, Fuzhou, China). The visualization was performed with a diaminobenzidine staining solution (DAB0031; Zhongshan Golden Bridge, Beijing, China). Last, the slices were stained with hematoxylin. Five random and different fields were captured for each slice. The positive cells were counted for each field using Trainable Weka Segmentation plugin (v3.3.4) in fiji/imagej [21].

For HE staining, the slices were stained with hematoxylin–eosin (HE). Then the histopathological score was assessed according to the previous report [22] by two independent professional pathologists.

### 2.5 Statistical analysis

Statistical analyses were performed in graphpad 8.0 (GraphPad Software, San Diego, CA, USA) with Student's two‐tail *t*‐test to analyze the two groups of continuous data otherwise specified. One‐way ANOVA was performed for assessing differences among three or more groups, and the Tukey's multiple comparisons was utilized. Kruskal–Wallis test and Dunn's multiple comparisons were used for ranked data. All tests performed were two‐tailed. There is a significant difference when *P* value < 0.05. Data are shown as the mean ± SEM.

## 3 Results

### 3.1 Subcutaneously inoculate tumor significantly changed gut microbiota composition

**Fig. 1:**
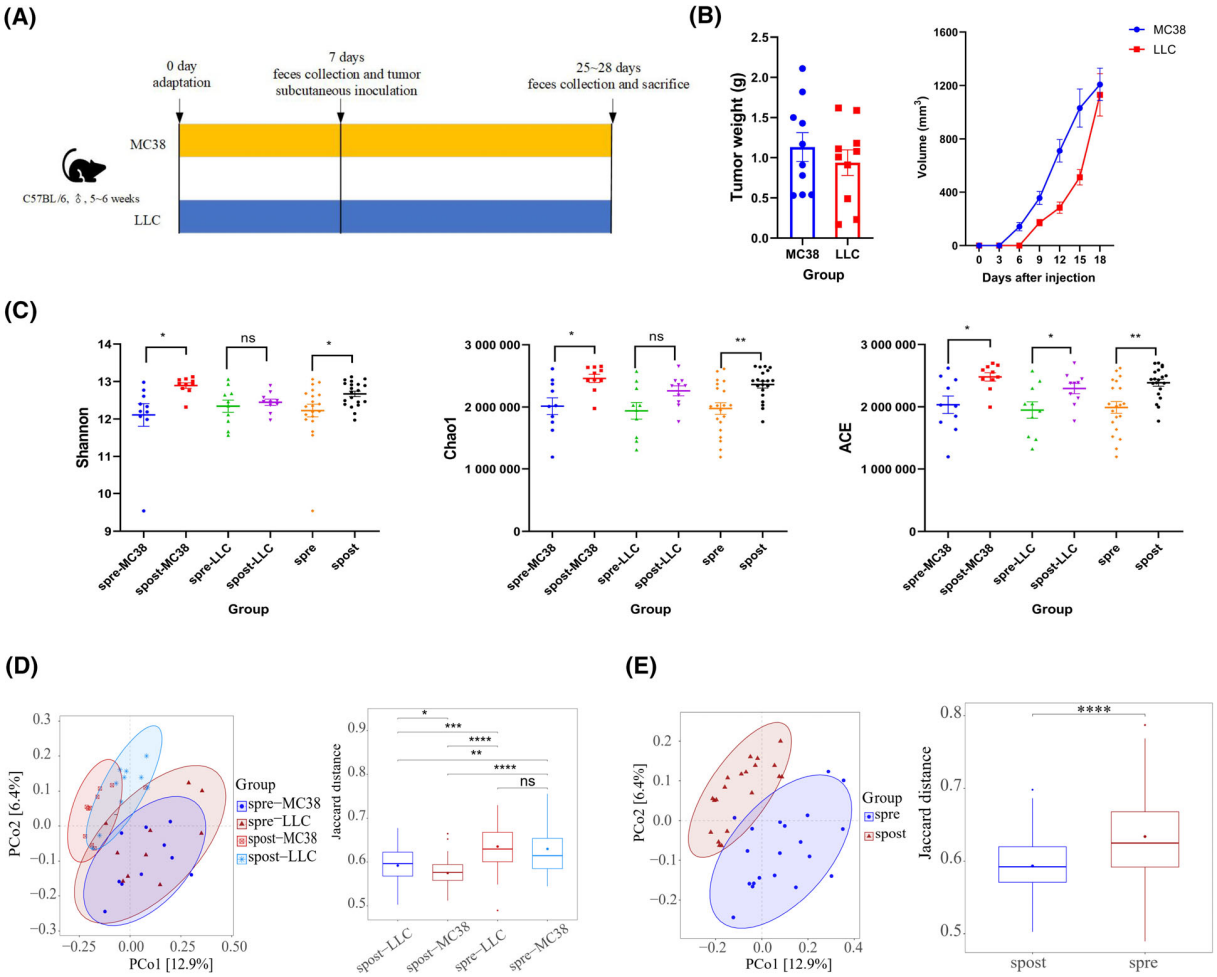
Subcutaneous tumor changed gut microbiota diversity. (A) Flowchart of subcutaneous inoculation of a tumor. (B) The weight (left) and volume (right) of the MC38 and LLC tumor. (C) Shannon, Chao1, and ACE indices among different groups. (D) PCoA of gut microbiota collected before (spre‐MC38 group) and after (spost‐MC38 group) MC38 tumor subcutaneous inoculation, as well as before (spre‐LLC group) and after (spost‐LLC group) LLC tumor subcutaneous inoculation, at the jaccard distance. (E) PCoA of spre (combination of spre‐MC38 and spre‐LLC groups) and spost (combination of spost‐MC38 and spost‐LLC groups) groups at the jaccard distance. *N* = 10 per group. Statistical significance was assessed by Student's *t* test (C, E) or Kruskal–Wallis test (D). Data are shown as the mean ± SEM. NS, not significant. *P < 0.05, **P < 0.01, ***P < 0.001, ****P < 0.0001.

The Subcutaneous tumor model was established and the fecal samples before and after tumor inoculation were collected. Two kinds of murine tumor cell lines were used in this study: the LLC (murine lung cancer cell line) and MC38 (murine colorectum cancer cell line) cell lines. The tumors and the colorectums were carefully resected 18 days after inoculation (Fig. 1A). Tumor volume and mice weight were measured every 3 days (Fig. 1B, Fig. S1). The fecal samples were collected before (spre‐LLC) and after (spost‐LLC) LLC tumor subcutaneous inoculation, meanwhile before (spre‐MC38) and after (spost‐MC38) MC38 tumor subcutaneous inoculation.

To investigate the gut microbiota composition, the metagenomic sequencing was applied to analyze the feces from spre‐LLC, spre‐MC38, spost‐LLC, and spost‐MC38 groups. To elaborate the landscape of tumor inoculation, the spre‐LLC and spre‐MC38 groups were combined as spre group, and spost‐LLC and spost‐MC38 groups were combined as spost group. As shown in Fig. 1C, the Shannon (kind of α‐diversity which describes the species diversity and evenness), Chao1, and ACE indices (another α‐diversity that describe the species richness) were increased after a tumor was inoculated in both the MC38 and LLC tumor, although the LLC tumor not reached the statistical threshold. We also found that compared with a pre‐inoculation group, Shannon, Chao1, and ACE indices in spost group were also increased. These hinted that subcutaneous inoculated tumors could increase the diversity of the gut microbiota. Principal coordinates analysis (PCoA) is another index that is usually used to describe the variance of taxonomic abundance between ecosystems (β‐diversity) [23]. Figure 1D shows that spre‐MC38 group and spre‐LLC group are almost overlapped, and spost‐MC38 group and spost‐LLC group have a large overlap. But spre‐MC38 and spre‐LLC have little overlap with spost‐MC38 and spost‐LLC. In accordance with the above results, spre has little overlap with spost (Fig. 1E). These revealed that tumor exists may change gut microbiota composition and different tumor types may exhibit the same pattern.

As age might be a confounding factor with tumor growth, an age‐matched experiment was conducted. Feces were collected from 5‐ and 8‐week‐old mice and underwent 16 s sequencing subsequently. No significant changes were observed both on α‐diversity and β‐diversity (*P* > 0.05, Figs S2 and S3). Therefore, age is not a dominant factor contributing to the gut microbiota changes during the tumor growth.

### 3.2 Subcutaneous inoculating tumor caused gut microbiota dysbiosis

**Fig. 2:**
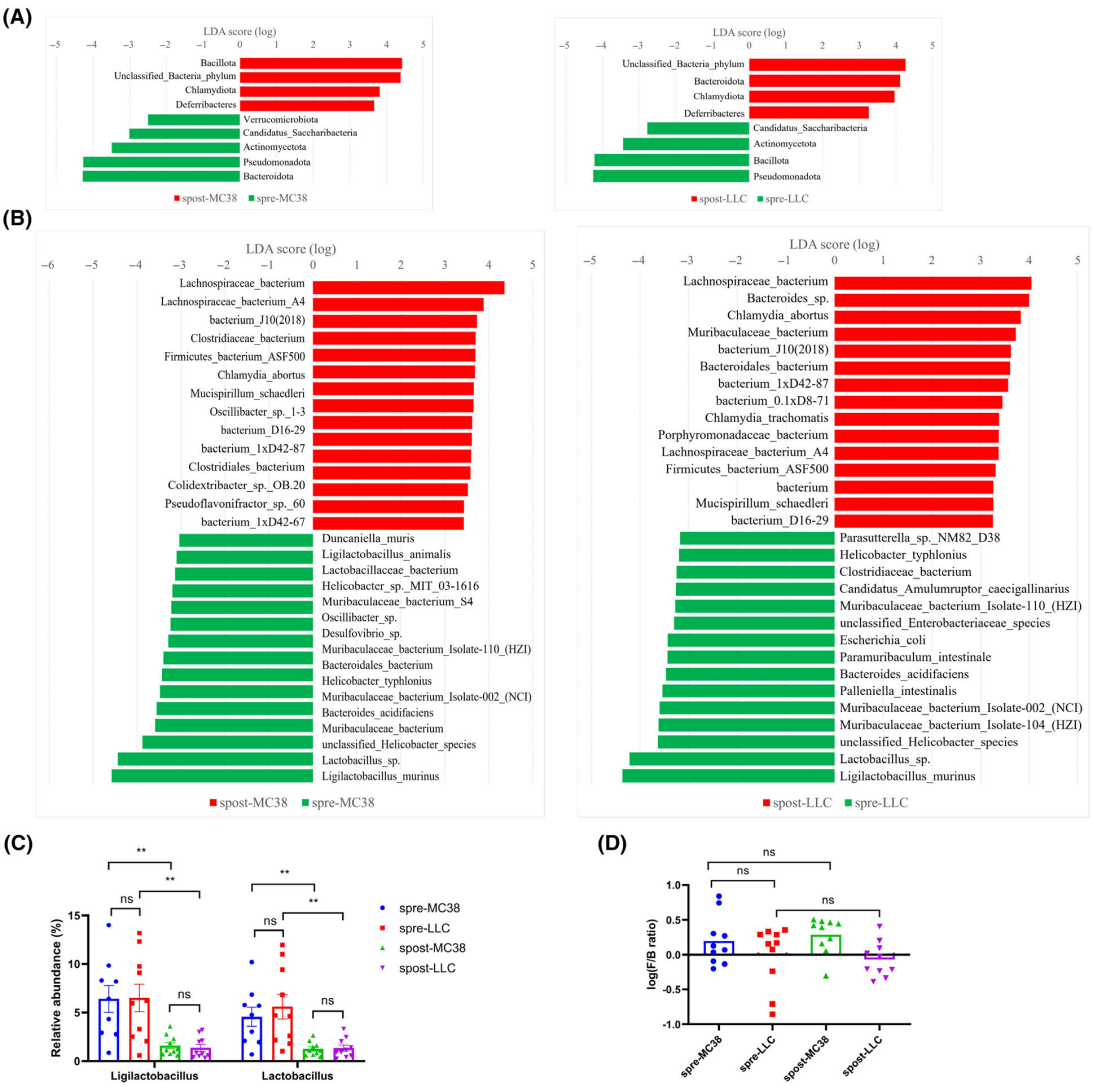
Subcutaneous tumor changed gut microbiota composition. LDA effect size (LEfSe) showed a significant difference in abundance (LDA score > 2) at phylum (A) and species level (B). (C) Distribution of *ligilactobacillus* and *Lactobacillus* among feces collected before (spre‐MC38 group) and after (spost‐MC38 group) MC38 tumor subcutaneous inoculation, as well as before (spre‐LLC group) and after (spost‐LLC group) LLC tumor subcutaneous inoculation. (D) Log ratio of *Firmicutes*/*Bacteroidetes* (F/B) among four groups. *N* = 10 per group. Statistical significance was assessed by Tukey's multiple comparisons in one‐way ANOVA. Data are shown as the mean ± SEM. NS, not significant. **P < 0.01.

Next, to investigate the extinct differences, the microbiota profile was compared among different groups at various taxon (Fig. S4). Linear discriminant analysis (LDA) effect size (LEfSe) was used to identify the difference distribution taxa between groups, and the LDA score > 2 and *P* value < 0.05 were set as threshold (Table S1). Figure 2A shows a significant difference in abundance of microbes at phylum level. Interestingly, most of the different microbiota were the same collected from both MC38 tumor and LLC tumor. *Chlamydiota* and *Deferribacteres* were mostly abundance in feces collected after tumor inoculation, while *Actinomycetota*, *Pseudomonadata*, and *Saccharibacteria* mainly existed in feces collected before tumor inoculation. However, *Bacteroidota* (also known as *Bacteroidetes*) and *Bacillota* (also known as *Firmicutes*) showed the contrast trends between MC38 tumor and LLC tumor. *Bacillota* was mainly abundance in feces collected from mice after MC38 inoculation, and *Bacteroidota* was abundance before MC38 inoculation. In contrast, *Bacillota* was abundance before LLC inoculation, and *Bacteroidota* was abundance after LLC inoculation. At species level, a total of 220 species between spre‐MC38 and spost‐MC38 groups, and 193 species between spre‐LLC and spost‐LLC groups were identified. When comparing the top15 most different abundance species, *Lachnospiraceae bacterium*, *Lachnospiraceae bacterium A4*, *bacterium J10(2018)*, *Firmicutes bacterium ASF500*, *Chlamydia abortus*, *Mucispirillum schaedleri*, *bacterium D16–29*, and *bacterium 1xD42–87* were signatures in both spost‐MC38 and spost‐LLC groups (Fig. 2B). Whereas a decreased abundance of *Muribaculaceae bacterium Isolate‐110 (HZI)*, *Helicobacter typhlonius*, *Muribaculaceae bacterium Isolate‐002 (NCI)*, *Bacteroides acidifaciens*, *unclassified Helicobacter species*, *Lactobacillus sp*., and *Ligilactobacillus murinus* were observed in both spost‐MC38 and spost‐LLC groups, comparing with their respective controls.

Interestingly, *Ligilactobacillus* and *Lactobacillus* were notably decreased after both the MC38 and LLC tumor inoculation (Fig. 2C). *Ligilactobacillus* [24, 25] and *Lactobacillus* [26, 27, 28] were also known as probiotics that take part in homeostasis and pathogenesis. Decreasing probiotics hinted at the gut dysbiosis of these tumor‐bearing mice. The higher ratio of *Firmicutes*/*Bacteroidetes* (F/B) is often observed in cancer, hypertension, and autism spectrum disorder patients [29, 30, 31]. However, no significant different ratio of F/B before and after tumor inoculation were noted (Fig. 2D).

### 3.3 Metastatic tumor could change gut microbiota composition and cause dysbiosis

**Fig. 3:**
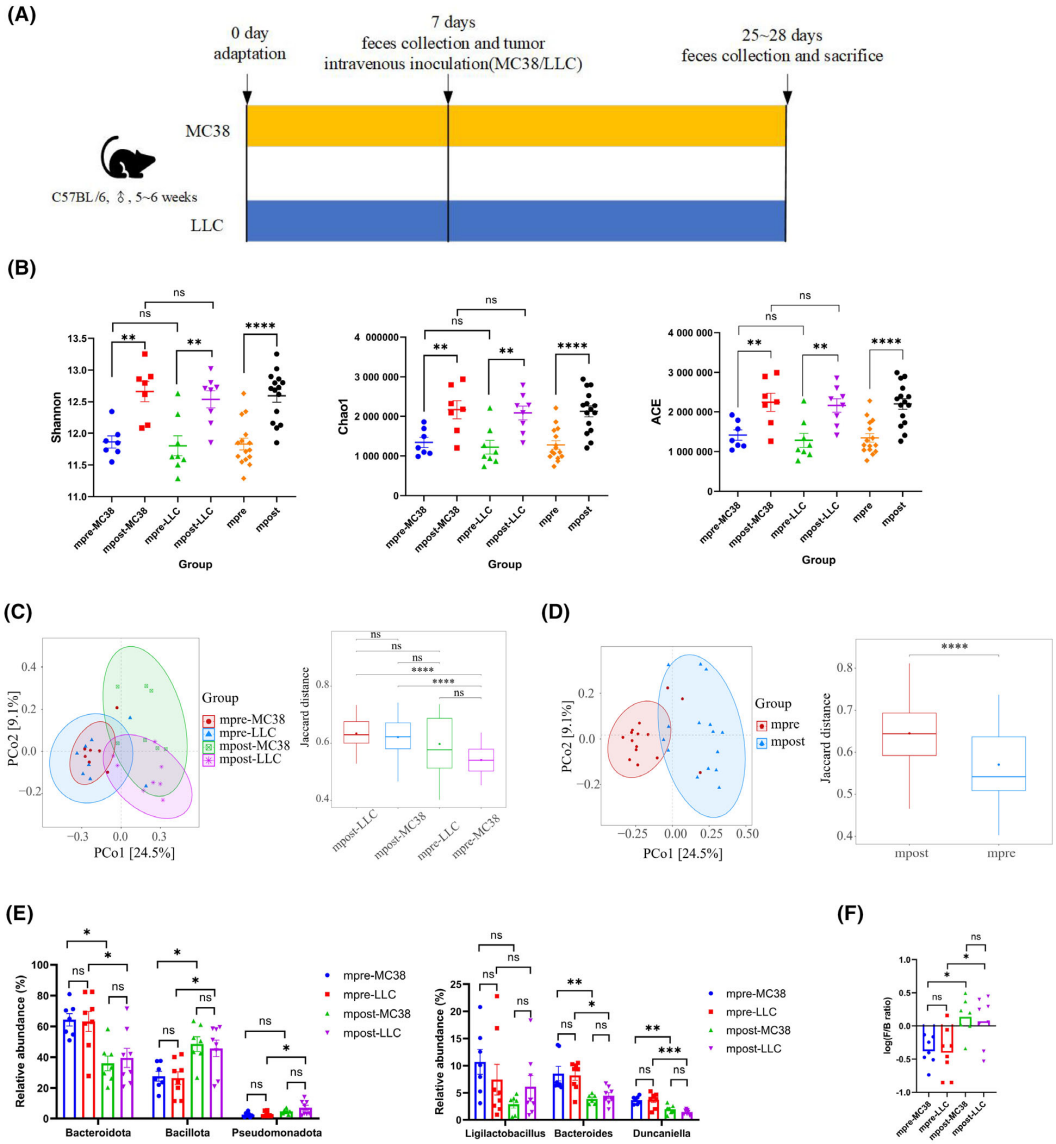
Metastatic tumors significantly altered the gut microbiota. (A) Flowchart of the construction of metastatic tumor model. (B)Shannon, Chao1, and ACE indices among different groups. (C) PCoA of gut microbiota collected before (mpre‐MC38 group) and after (mpost‐MC38 group) MC38 tumor intravenous inoculation, as well as before (mpre‐LLC group) and after (mpost‐LLC group) LLC tumor intravenous inoculation, at the jaccard distance. (D) PCoA of mpre (combination of mpre‐MC38 and mpre‐LLC groups) and mpost (combination of mpost‐MC38 and mpost‐LLC groups) groups at the jaccard distance. (E) Distribution of *Bacteroidota*, *Bacillota*, and *Pseudomonadota* (left) among four groups as well as distribution of *Ligilactobacillus*, *Lactobacillus*, and *Duncaniella* (right) among four groups. (F) Log ratio of *Firmicutes*/*Bacteroidetes* (F/B) among four groups. *N* = 7 for MC38 group and *N* = 8 for LLC group. Statistical significance was assessed by Student's *t* test (B, D) or Tukey's multiple comparisons in one‐way ANOVA (E, F) or Kruskal–Wallis test (C). Data are shown as the mean ± SEM. NS, not significant. *P < 0.05, **P < 0.01, ***P < 0.001, ****P < 0.0001.

Metastasis often occurs at the terminal of the tumor. As metastatic tumor has distinct pathogenesis and treatment strategies with carcinoma *in situ*, we wonder to know whether metastatic tumors could change the gut microbiota. Mice were injected with MC38 or LLC tumor cells intravenously and the fecal samples were collected before (mpre‐LLC) and after (mpost‐LLC) LLC cell intravenous injection, meanwhile before (mpre‐ MC38) and after (mpost‐MC38) MC38 cell intravenous injection (Fig. 3A). Consistent with previous report [32, 33, 34], MC38 and LLC cells were successfully metastasized to lung tissue but not colorectum (Fig. S5). The feces from mpre‐LLC, mpre‐MC38, mpost‐LLC, and mpost‐MC38 groups were underwent metagenomic sequencing. The mpre‐LLC and mpre‐MC38 groups were combined as mpre group, and mpost‐LLC and mpost‐MC38 groups were combined as mpost group. Compared with the feces collected from mice before tumor inoculation, the Shannon, Chao1, and ACE indices were markedly increased after MC38 or LLC inoculation (Fig. 3B). The mpost also had higher α‐diversity than mpre. Interestingly, these results were consistent with previous subcutaneous tumors, hinting that both subcutaneous and metastatic tumors could enrich the gut microbiota. PCoA showed that mpre‐MC38 group fully overlapped with mpre‐LLC, but had less overlap with mpost‐MC38 (Fig. 3C). Combined analysis also showed that less overlap between mpre group and mpost group (Fig. 3D). These showed that injected tumor through tail vein could also change gut microbiota without colorectum metastasis.

Then, the gut microbiota abundance distribution at various taxa were assessed (Fig. S6). The *Bacteroidota* was markedly enriched in mpre‐MC38 and mpre‐LLC groups at phylum level, and *Bacillota* and *Pseudomonadota* were mainly dominate in mpost‐MC38 and mpost‐LLC groups (Fig. 3E, left). Notably increased F/B ration was observed in both mpost‐MC38 and mpost‐LLC groups (Fig. 3F). At genus level, LDA (Table S2) showed that *Ligilactobacillus*, *Bacteroides*, and *Duncaniella* were the biomarkers for pre‐MC38 and pre‐LLC groups, and Tukey's analysis in one‐way ANOVA showed that the relative abundance of *Bacteroides* and *Duncaniella* was markedly reduced after MC38 or LLC inoculation (Fig. 3E, right). *Bacteroides* was found in abundance in healthy controls and positively related with treatment outcomes [29, 35, 36]. *Duncaniella* existed in healthy mice gut and protecting mice from colitis [37]. At species level, a total of 263 species between mpre‐MC38 and mpost‐MC38 groups, and 251 species between mpre‐LLC and mpost‐LLC groups were identified according to LEfSe analysis with the threshold of *P* < 0.05 and the LDA score > 2 (Fig. S7). Interestingly, 9 species of the 15 top abundance species were same in mpost‐MC38 and mpost‐LLC groups, as well as 9 species of the 15 top abundance species were common microbial signatures in mpre‐MC38 and mpre‐LLC groups. These hinting metastatic MC38 and LLC tumors may make similar functions to gut microbiota. Taken together, metastatic tumors could also change gut microbiota and cause gut dysbiosis.

### 3.4 Metabolic functions of gut microbiota shifted by subcutaneous and metastatic tumor

As tumor markedly changed gut microbiota composition, we wonder to know functional features shifted by tumor. KEGG pathway analysis were performed to compare differently enriched metabolic modules among groups based on the LEfSe analysis.

**Fig. 4:**
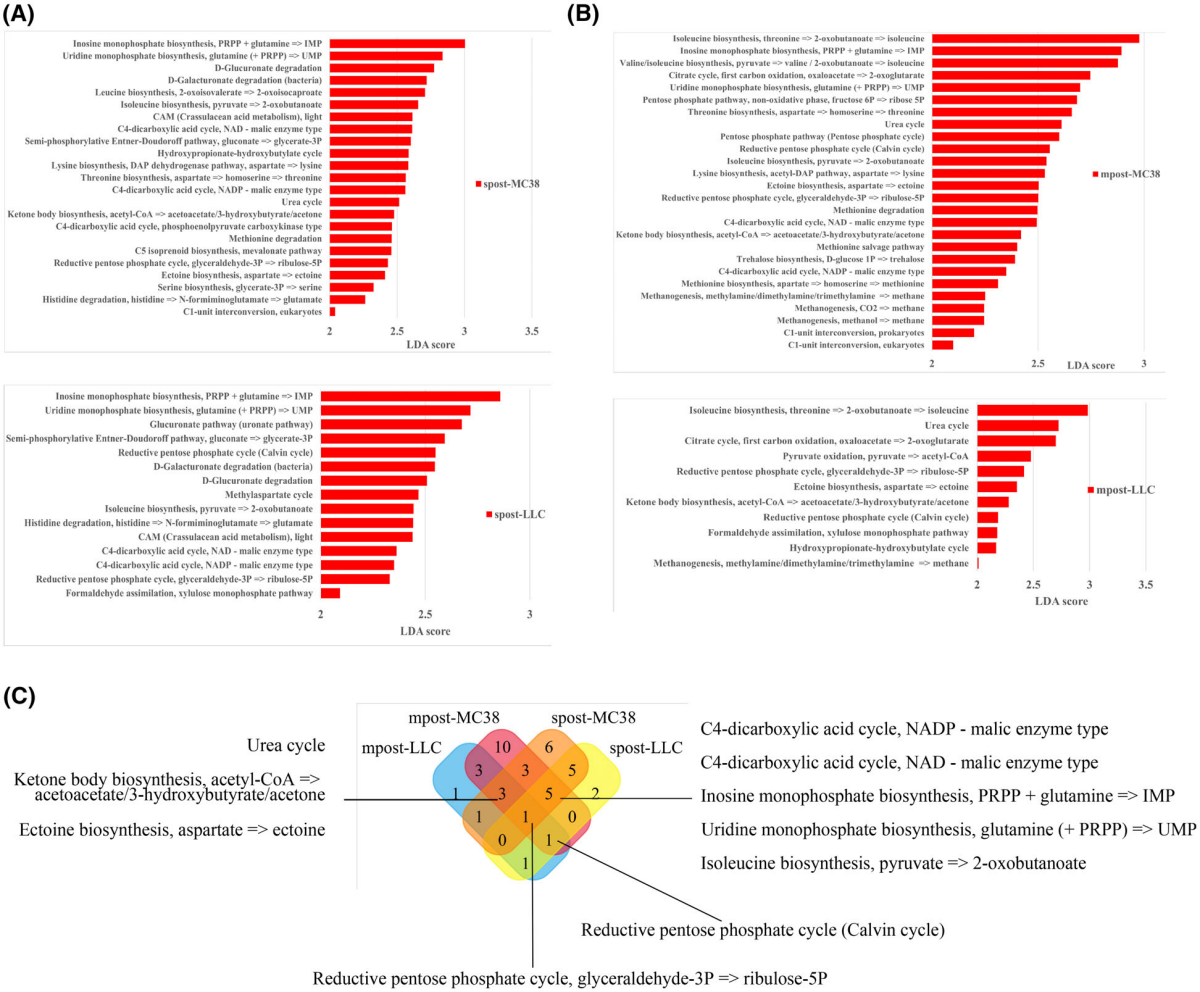
Enriched KEGG pathway modules of metabolic function of gut microbiota in post tumor inoculation groups. (A) Enriched modules in gut microbiota collected after MC38 (spost‐MC38 group) and LLC (spost‐LLC group) tumor subcutaneous inoculation compared with their respective pre‐inoculation groups. (B) Enriched modules in gut microbiota collected after MC38 (mpost‐MC38 group) and LLC (mpost‐LLC group) tumor intravenous inoculation compared with their respective pre‐inoculation groups. (C) Veen diagram of enriched modules among four groups.

Among the subcutaneous groups, a total of 48 KEGG modules were significantly differently enriched between spre‐MC38 and spost‐MC38 groups, as well as 26 differently enriched KEGG modules between spre‐LLC and spost‐LLC groups (Fig. 4A and Fig. S8A). Modulates enriched in spost‐MC38 group mainly included C4‐dicarboxylic acid cycle, reductive pentose phosphate cycle, amino acid biosynthesis, isoprenoid biosynthesis, purine degradation, UMP and IMP biosynthesis, and ketone body biosynthesis. Meanwhile, cofactor and vitamin biosynthesis, drug resistance, nucleotide biosynthesis, leucine degradation, fatty acid, and lipid biosynthesis and were markedly enriched in spre‐MC38 groups. In the LLC inoculation groups, C4‐dicarboxylic acid cycle, reductive pentose phosphate cycle, amino acid metabolism, UMP, and IMP biosynthesis were enriched modules in spost‐group, while drug resistance, ribonucleotide biosynthesis, lipopolysaccharide, and lipid biosynthesis were depleted.

Among the metastasis groups, 68 KEGG modules were enriched between mpre‐MC38 and mpost‐MC38 groups, and 26 KEGG modules were enriched between mpre‐LLC and mpost‐LLC groups (Fig. 4B and Fig. S8B). Amino acid biosynthesis, IMP and UMP biosynthesis, methanogenesis, ketone body biosynthesis, dicarboxylic acid cycle, pentose phosphate pathway, and reductive pentose phosphate cycle modules were elevated in mpost‐group, while cofactor and vitamin biosynthesis, drug resistance, fatty acid, lipid biosynthesis, lipopolysaccharide biosynthesis, beta‐Oxidation, glycolysis, and citrate cycle modules were decreased. In the LLC metastasis groups, reductive pentose phosphate cycle, ketone body biosynthesis, methanogenesis, isoleucine biosynthesis, urea cycle, and ectoine biosynthesis were elevated in mpost‐LLC group, while cofactor and vitamin biosynthesis, beta‐oxidation, lysine biosynthesis, keratan sulfate degradation modules were mainly enriched in the mpre‐LLC group.

Interestingly, among the four postinoculation groups, reductive pentose phosphate cycle, urea cycle, ketone body biosynthesis, ectoine biosynthesis, C4‐dicarboxylic acid cycle, isoleucine biosynthesis, IMP, and UMP biosynthesis were the common features for more than three groups (Fig. 4C). However, cofactor and vitamin biosynthesis were mainly enriched among pre‐inoculation groups. Indicting the cofactor and vitamin biosynthesis were deficiency after tumor inoculation.

### 3.5 Different tumor types and stages partially share the same effect pattern with gut microbiota

**Fig. 5:**
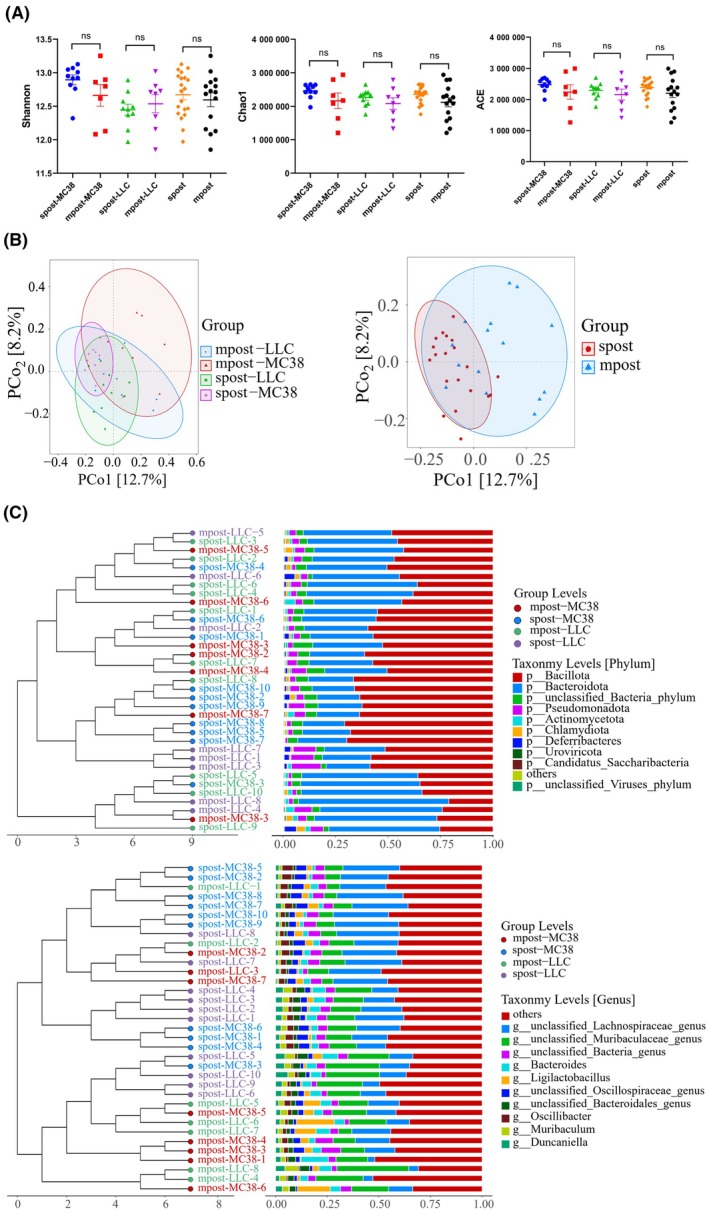
The similarity between subcutaneous and metastatic tumors. (A) The Shannon, Chao1, and ACE indices among different groups. (B) PCoA of gut microbiota collected after MC38 (spost‐MC38 group, *n* = 10) and LLC (spost‐LLC group, *n* = 10) subcutaneous inoculation, as well as after MC38 (mpost‐MC38 group, *n* = 7) and LLC (mpost‐LLC group, *n* = 8) intravenous inoculation, at jaccard distance (left). PCoA of spost (combination of spost‐MC38 and spost‐LLC groups) and mpost (combination of mpost‐MC38 and mpost‐LLC groups) groups at jaccard distance (right). (C) Composition of individuals collected after tumor inoculated at phylum (upper) and genus level (lower). Statistical significance was assessed by Student's t test. Data are shown as the mean ± SEM. NS, not significant.

Due to both of the subcutaneous and metastatic tumors could alter the gut microbiota, whether this changed pattern had the similarity remains interesting. We found that the α‐diversity between subcutaneous and metastasis tumor groups were similar (*P* > 0.05) as detected by Shannon, Chao1, and ACE indices (Fig. 5A). PCoA also showed the large overlap was found between spost‐MC38, spost‐LLC, mpost‐MC38, and mpost‐LLC groups (Fig. 5B, left). And also, spost and mpost groups almost totally overlapped (Fig. 5B, right), indicting the similarity of the effects of subcutaneous and metastatic tumors to gut microbiota. Based on the former top 15 most abundance species, Veen diagram showed the *Lachnospiraceae bacterium* was the common signature for spost‐MC38, spost‐LLC, mpost‐MC38, and mpost‐LLC, while *Muribaculaceae bacterium Isolate‐110 (HZI)*, *Ligilactobacillus murinus* and *B. acidifaciens* were the common features for spre‐MC38, spre‐LLC, mpre‐MC38, and mpre‐LLC (Fig. S9). We then cluster and compare the gut microbiota composition at different taxons. As shown in Fig. 5C, not all of the samples from the same group were clustered together at phylum and genus level, hinting no marked differences were found across each group. The composition of the microbiota was also apparently similar. Taken together, these data suggest that the different tumor types (MC38 and LLC) and different tumor stages (subcutaneous tumor and metastatic tumor) partially share the same effects on gut microbiota.

### 3.6 The structure of colorectum was not damaged after tumor inoculation

**Fig. 6:**
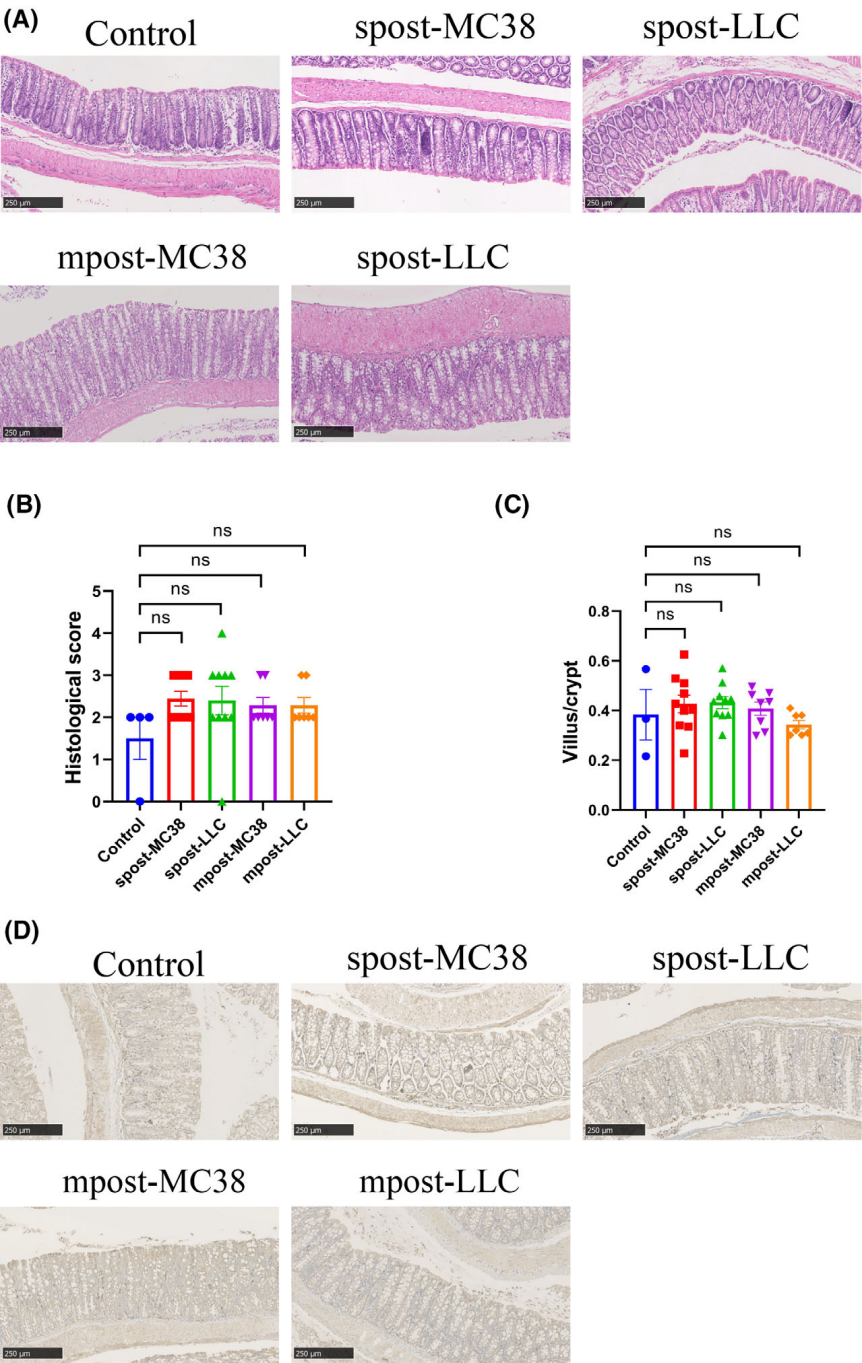
No marked colorectum mucosa injury was observed in tumor‐bearing mice. (A) Representative HE staining images of colorectum collected from control group (*n* = 4), MC38 (spost‐MC38 group, *n* = 10), and LLC (spost‐LLC group, *n* = 10) subcutaneous inoculation groups, as well as from MC38 (mpost‐MC38 group, *n* = 7) and LLC (mpost‐LLC group, *n* = 8) intravenous inoculation groups. Histological score (B) and villus/crypt (C) among different groups. (D) ZO‐1 expression levels in the colorectum of different groups were visualized by immunohistochemistry. Scale bar = 250 μm. Statistical significance was assessed by Dunn's multiple comparisons in Kruskal–Wallis test (B) or Tukey's multiple comparisons in one‐way ANOVA (C). Data are shown as the mean ± SEM. NS, not significant

As feces were closely related to the contains in colorectum, we then scored the colorectum mucosa by two independent pathologists according to one previous standard [22]. Interestingly, the pathologists reported no significant inflammation and mucosa injury, such as atrophy and abrasion, were observed within colorectum collected after tumor inoculation (Fig. 6A). Histological grading also showed no significant difference among control, spost‐MC38, spost‐LLC, mpost‐MC38, and mpost‐LLC groups (Fig. 6B). Meanwhile, the villus/crypt height ratio also showed no significant difference among groups (Fig. 6C). We then tested the expression of tight junction protein Zona occludens 1 (ZO‐1) in the colorectum tissue. Consistent with the above results, these groups exhibited a similar expression level of ZO‐1 (Fig. 6D). These results indicated that the gut dysbiosis induced by subcutaneous or metastatic tumor did not disrupt the structure of colorectum.

### 3.7 Transplanting feces from pre‐inoculation mice reduced tumor growth

**Fig. 7:**
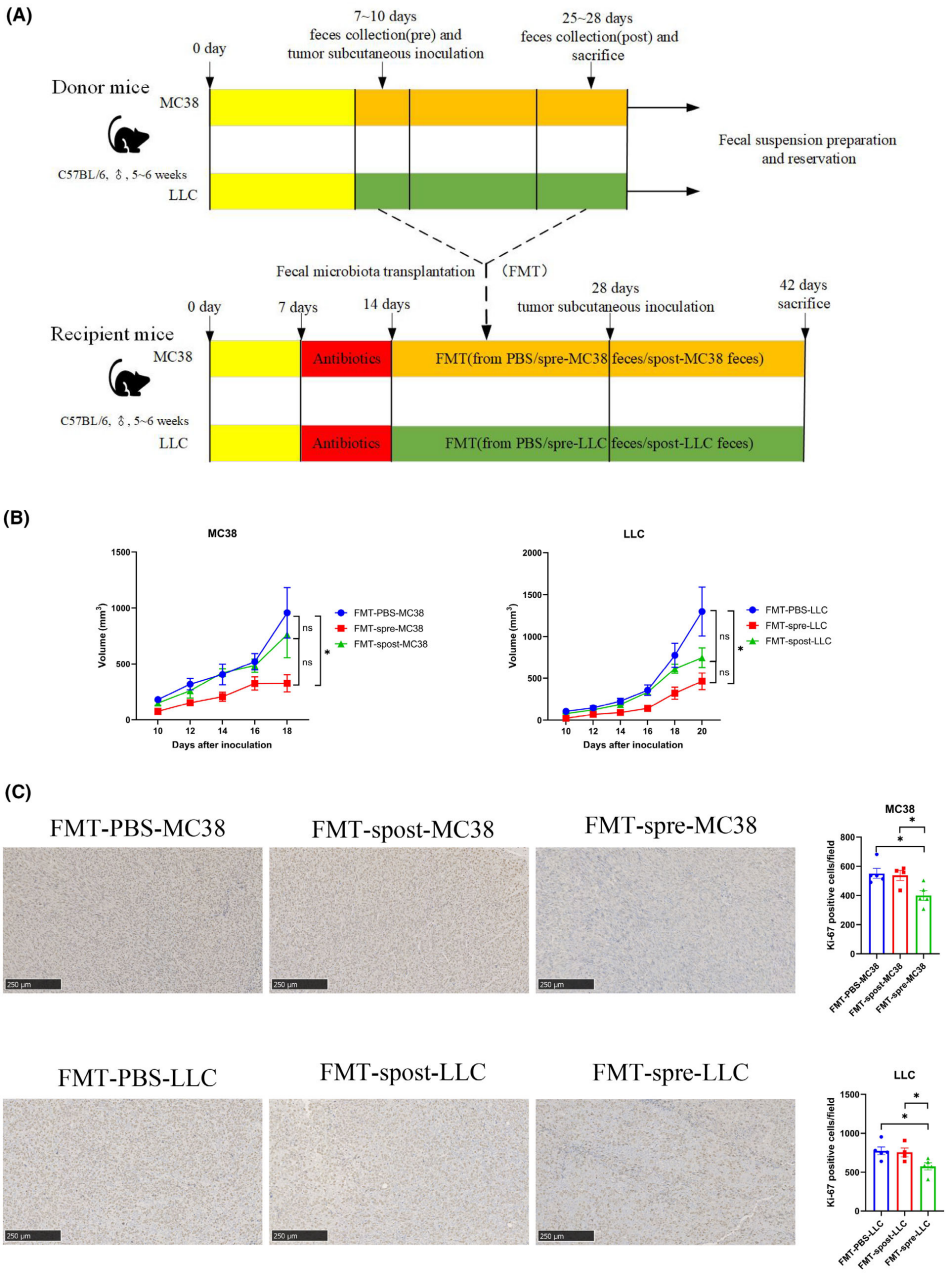
FMT of feces from preinoculation mice reduced tumor growth. (A) Flowchart of the transplantation of feces from mice before (FMT‐spre‐MC38) and after (FMT‐spost‐MC38) inoculation of MC38 tumor, as well as before (FMT‐spre‐LLC) and after (FMT‐spost‐LLC) inoculation of LLC tumor to recipient mice, respectively. (B) Tumor volume of MC38 (left) and LLC (right) was measured every 2 days. (C) Immunohistochemistry of tumor tissue for Ki‐67 expression (scale bar = 250 μm) among different groups. One mouse in the FMT‐spost‐MC38 group and one in the FMT‐spost‐LLC group died during gavage process. Statistical significance was assessed by Tukey's multiple comparisons in one‐way ANOVA. Data are shown as the mean ± SEM. *N* = 5 per group. NS, not significant. *P < 0.05

As the gut microbiota changed a lot after tumor inoculation both subcutaneous and intravenous, we wonder to know whether these changed feces could influence tumor growth. All recipient subjects orally taken broad‐spectrum antibiotics (ampicillin, colistin, neomycin, vancomycin) 7 days before FMT. Then FMT was conducted with feces collected from donor mice before or after tumor inoculation (Fig. 7A). The fecal suspensions were orally injected into recipient mice 2 weeks prior to the tumor inoculation. The recipient mice inoculated MC38 cells and transplanted PBS, pre‐MC38 feces, and post‐MC38 feces were marked as FMT‐PBS, FMT‐spre‐MC38, and FMT‐spost‐MC38 groups, respectively. So did the recipient mice inoculated LLC tumor cells. Compared with the FMT‐PBS group, transplanting feces from pre‐inoculation mice significantly reduced the MC38 and LLC tumor growth (Fig. 7B, Fig. S10, *P* < 0.05). Compared with the FMT‐spost group, transplanting feces from pre‐inoculation group also showed a trend of shrinking tumor volume, though not reaching the statistic threshold (*P* > 0.05). Consistent with the above results, immunohistochemistry showed that the number of Ki‐67‐positive tumor cells were downregulated in FMT‐spre‐MC38 group and FMT‐spre‐LLC group (Fig. 7C). These results suggested that transplanting feces from pre‐inoculation mice could prevent tumor growth, hinting probiotics existing within the feces from these mice.

## 4 Discussion

Numerous studies have demonstrated that gut microbiota, the largest source of microorganisms in the body, plays roles in the development of a series of illnesses, including aging, psychological illness, and cancer [12, 38, 39]. Douglas Hanahan [40] also added polymorphic microbiomes as an emerging hallmark of cancer. The direct functions of gut microbiota to colorectum cancer have been elucidated recently. Some specific species may play roles through mutating epithelium cells, interacting with the immune system or secreting functional metabolite [39, 41]. The indirect functions of gut microbiota to the tumor out of the gut are still under exploring. *Enterococcus hirae* can translocate to the secondary lymphoid organs, which stimulate the immune response and reduce distant cancer growth [42]. Despite the above inspiring findings, the causality of gut microbiota and cancer is still unknown. In this study, we aim to figure out the alterations of gut microbiota shifted by tumors. We systematically analyzed the mice gut microbiota with distinct tumor types and tumor inoculation ways. We found both subcutaneous and intravenous inoculating tumors could markedly change gut microbiota composition and cause gut dysbiosis. And these changed patterns were partially same across different tumor types and tumor inoculation ways. Meanwhile, no significant damages of colorectum mucosa were found whether tumor inoculation. Transplanting feces from pre‐inoculation mice could reduce tumor growth.

Shannon, Chao1, and ACE are commonly used to assay community α‐diversity. The relationship between α‐diversity and cancer is controversial. Some studies observed elevated α‐diversity in cancer patients [43, 44]. However, recent studies found that patients with cancer had lower α‐diversity than health controls [45, 46, 47, 48]. Conversely, others concluded similar α‐diversity between the cancer and healthy groups [4, 31, 49]. In this study, the α‐diversity (Shannon, Chao1, and ACE) of feces was elevated in tumor‐bearing mice, regardless of tumor type and tumor inoculating ways. This contradiction may be due to the type and stage of the tumor, and the sequencing method used in the study. The causality between cancer and feces α‐diversity should be explored further as the higher α‐diversity often hints at more favorable clinical outcome [23, 29, 50].

Stable gut microbiota composition is considered crucial to health. Gut dysbiosis has been proved involved in autism spectrum disorder, diabetes mellitus, and nonalcoholic fatty liver disease [51, 52, 53]. We found the gut microbiota composition changed a lot before and after tumor inoculation whether subcutaneous or intravenous (Figs 1D,E, 2A,B, and 3C–F, Figs S4, S6, and S7). *Bacillota* and *Bacteroidota* were the main abundance phyla in mice gut, and the increased ratio of F/B often indicates gut dysbiosis [54, 55]. A recent study considered an elevated F/B ratio as a risk factor of breast cancer [56]. We found the markedly increased F/B ratio after intravenously injected tumor, rather than subcutaneous tumor (Figs 2D and 3F). This hinted more severe gut dysbiosis caused by metastatic tumors. Meanwhile, we also found some probiotics, such as *Ligilactobacillus*, *Lactobacillus*, *Bacteroides*, and *Duncaniella* were decreased after tumor was inoculated. We provided evidence that the changed gut microbiota was caused by the existence tumor. Some of these specific species were found involved in tumorigenesis and treatment [57, 58]. Long et al. also reported the causal relationship between *Lactobacillales* and lung cancer [9]. Interestingly, *Bacteroides* was decreased in cancer patients and positively correlated with treatment outcome [36]. Preclinical study also demonstrated the existing of *Bacteroides* was crucial for the efficiency of immunotherapy [59]. We give clues that the decrease of *Bacteroides* in cancer patients' feces may be the function of tumor. Taken together, we provided evidence that the changed gut microbiota was caused by the existing tumor. The specific mechanism underlying the crosstalk between gut microbes and tumors still remains unclear. We speculate that certain particular factors released by the tumor or the disrupted immune system may be responsible for the shifted gut microbiota. The detailed mechanisms still need further exploration.

When tumor cells invade the mucosa and disseminate to distant organs, metastasis occurs, often at the ultimate stage of the tumor. As metastatic tumor has distinct pathogenesis and treatment strategies with subcutaneous tumors, whether metastatic tumors and subcutaneous tumors exhibit similar influence on gut microbiota needed further exploration. We found certain similarities of gut microbiota profile exist between subcutaneous tumor and metastatic tumor‐bearing mice. They have the similar gut microbiota diversity (Fig. 5A) and PCoA also showed large overlapping area (Fig. 5B). Taken together, these results imply that distinct tumor types and stages may share the same effects to gut microbiota.

Alterations in gut microbiota composition commonly transform microbial metabolic function pathways. In the lipid metabolism, metabolic function switches from lipid and lipopolysaccharide biosynthesis in pre‐inoculation group to ketone body biosynthesis in postinoculation group (spost‐MC38, mpost‐MC38, and mpost‐LLC groups). Ketone body could not be utilized by cancer cells and therefore restricted them from growth [60]. Lipopolysaccharide could accelerate breast cancer growth form commensal bacterium [61]. In this aspect, tumor‐shifted gut microbiota to produce more ketone bodies and less lipopolysaccharide, which could restrict their growth. Another concerned metabolic module is deficient of cofactor and vitamin biosynthesis, including menaquinone, ascorbate, NAD, pantothenate, tetrahydrofolate, and biotin. These cofactors and vitamins were essential to human health some of which were seen as tumor suppressors [62, 63]. These may contribute to the cancer patients' cachexia and resistance to treatment.

Ileopathy was observed within tumor‐bearing mice, and *E. hirae* was found translocated to spleen from small intestine, affecting the efficiency of chemotherapy [10, 42]. However, the fecal samples, one of the easily and widely collected specimens in clinic, were thought closely related with colorectum [64]. We wonder whether the gut dysbiosis, caused by subcutaneous and metastatic tumors, could disrupt the structure of colorectum. Surprisingly, both the subcutaneous and metastatic tumors did not affect the structure of colorectum. Indicating that cancer may not directly cause colorectum mucosa injury. However, in clinics, the usage of certain drugs, such as antibiotics [4], could disrupt the structure of colorectum and such complex situations may perplex the relationship between cancer and gut microbiota. Further study is worthy to be conducted to explore the tumor‐microbiota crosstalk in such complex situations.

Furthermore, the gut dysbiosis caused by cancer could affect tumor growth to some extent. Transplanting feces from SPF mice to germ‐free mice could reduce tumor metastasis, and disrupting gut microbiota with antibiotics could promote tumor growth, highlighting the crucial role of gut microbiota to cancer [4, 34]. Indeed, compared with PBS transplantation group, transplanting feces from pre‐inoculation mice could significantly reduce tumor growth and ki‐67 expression. Indicting feces from pre‐inoculation mice may contain probiotics against the tumor. Meanwhile, compared with FMT‐PBS group, transplanting feces from postinoculation mice did not show increased tumor growth, but showed a trend of increased growth when compared with FMT‐spre MC38 and FMT‐spre‐LLC groups, respectively. Due to feces containing multiple microbes, we speculate that the promoting or inhibiting tumor factors may be obscured by the bystanders. It's necessary to explore the effects of single microbe from the pre‐FMT or post‐FMT group.

## 5 Conclusions

In summary, we provide novel insights on the crosstalk between gut microbiota and tumors. We demonstrate that tumors could cause gut dysbiosis and metabolic imbalance, characterized by the reduction of *Ligilactobacillus* and *Lactobacillus in* subcutaneous tumors, and *Bacteroides* and *Duncaniella* in metastatic tumors, without injury of colorectum mucosa. Tumor‐shifted gut microbiota produced more ketone body and less lipopolysaccharide. Meanwhile, transplanting feces from pre‐inoculation mice could reduce tumor growth.

## Conflict of interest

The authors declare no conflict of interest.

## Author contributions

SZ and SY conceived and designed the study. SZ, BY, HW, and YC established the murine tumor model. SZ, HW, and JN collected the sample and conducted the experiments. SZ, HW, DH, YD, and CY analyzed the data. SZ, HW, and SY drafted and revised the manuscript. All authors have read and approved the final manuscript.

## Supporting information


**Fig. S1.** Subcutaneous tumor.
**Fig. S2.** Shannon, Chao1, and ACE indices of gut microbiota from 5‐week‐old and 8‐week‐old mice.
**Fig. S3.** PCA analysis of gut microbiota from 5‐week‐old and 8‐week‐old mice.
**Fig. S4.** The gut microbiota composition (top 10) at phylum and genus level of subcutaneous tumor.
**Fig. S5.** Metastatic tumor.
**Fig. S6.** The gut microbiota composition (top 10) at phylum and genus level of metastatic tumor.
**Fig. S7.** The top 15 histograms of LDA coupled with effective size between mpost‐MC38 and mpre‐MC38 and between mpost‐LLC and mpre‐LLC.
**Fig. S8.** Enriched KEGG pathway modules of metabolic function of gut microbiota in pre‐inoculation groups.
**Fig. S9.** Veen diagram of top 15 most abundance species.
**Fig. S10.** Image of tumor from different groups inoculated MC38 or LLC in FMT.

**Table S1.** LEfSe analysis of difference distribution taxa between subcutaneous tumor.

**Table S2.** LEfSe analysis of difference distribution taxa between metastatic tumor.

## Data Availability

Whole metagenomic sequence data generated during process are deposited at the National Center for Biotechnology Information (NCBI) in Sequence Read Archive (SRA) under the BioProject accession number PRJNA1084102.
