# Supplementary material for: Crosstalk between gut microbiota and tumor: tumors could cause gut dysbiosis and metabolic imbalance
Source: Mol Oncol. 2024 Nov 26;19(6):1707–24. doi: 10.1002/1878-0261.13763 (PMC12161466; doi:10.1002/1878-0261.13763)
Supplement: Supplementary file 1 — Fig. S1. Subcutaneous tumor. Fig. S2. Shannon, Chao1, and ACE indices of gut microbiota from 5‐week‐old and 8‐week‐old mice. Fig. S3. PCA analysis of gut microbiota from 5‐week‐old and 8‐week‐old mice. Fig. S4. The gut microbiota composition (top 10) at phylum and genus level of subcutaneous tumor. Fig. S5. Metastatic tumor. Fig. S6. The gut microbiota composition (top 10) at phylum and genus level of metastatic tumor. Fig. S7. The top 15 histograms of LDA coupled with effective size between mpost‐MC38 and mpre‐MC38 and between mpost‐LLC and mpre‐LLC. Fig. S8. Enriched KEGG pathway modules of metabolic function of gut microbiota in pre‐inoculation groups. Fig. S9. Veen diagram of top 15 most abundance species. Fig. S10. Image of tumor from different groups inoculated MC38 or LLC in FMT. [file MOL2-19-1707-s002.pdf]

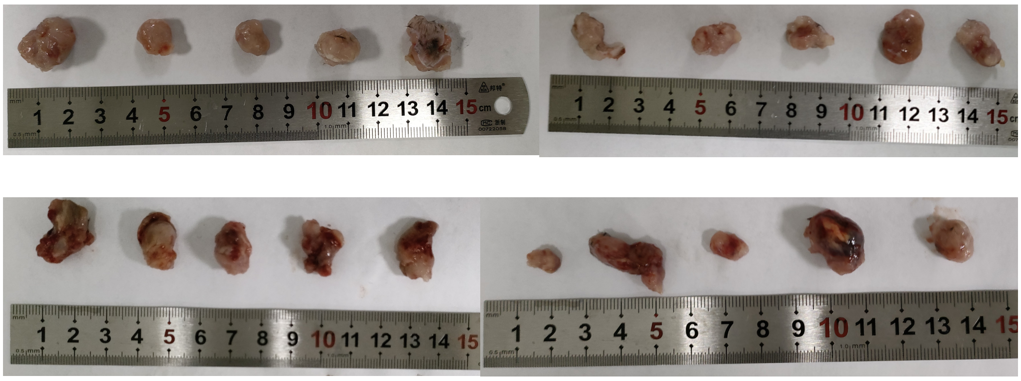

MC38

LLC

Supplementary figure 1. Subcutaneous tumor

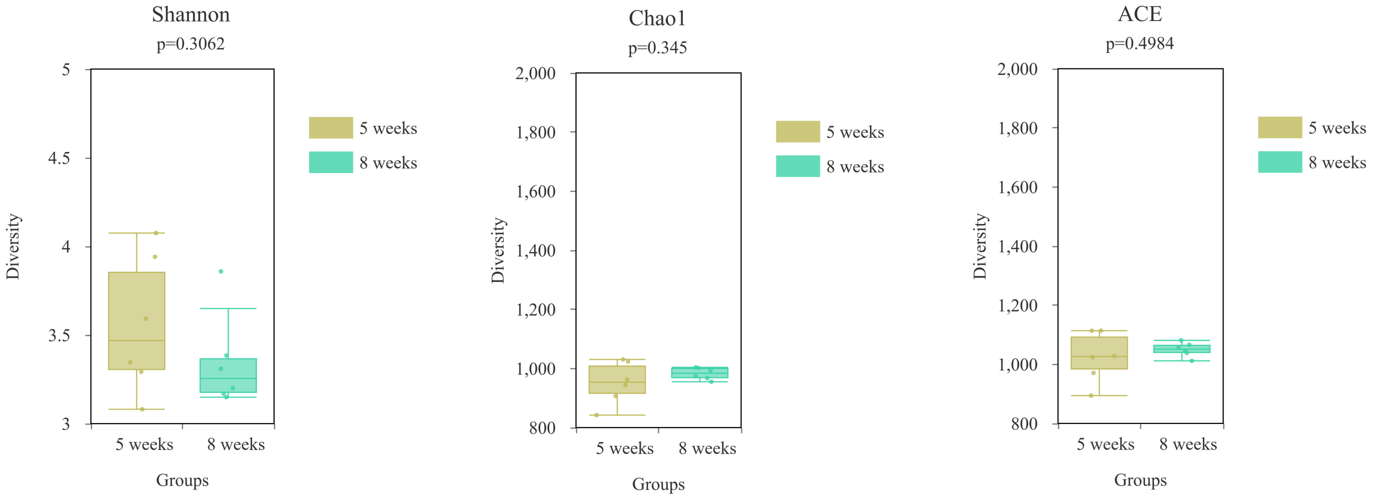

Supplementary figure 2. Shannon, Chao1 and ACE indices of gut microbiota from 5-weeks-old and 8-weeks-old mice. P value was calculated using Student's t test. Data are shown as the mean ± SEM. N = 6 per group.

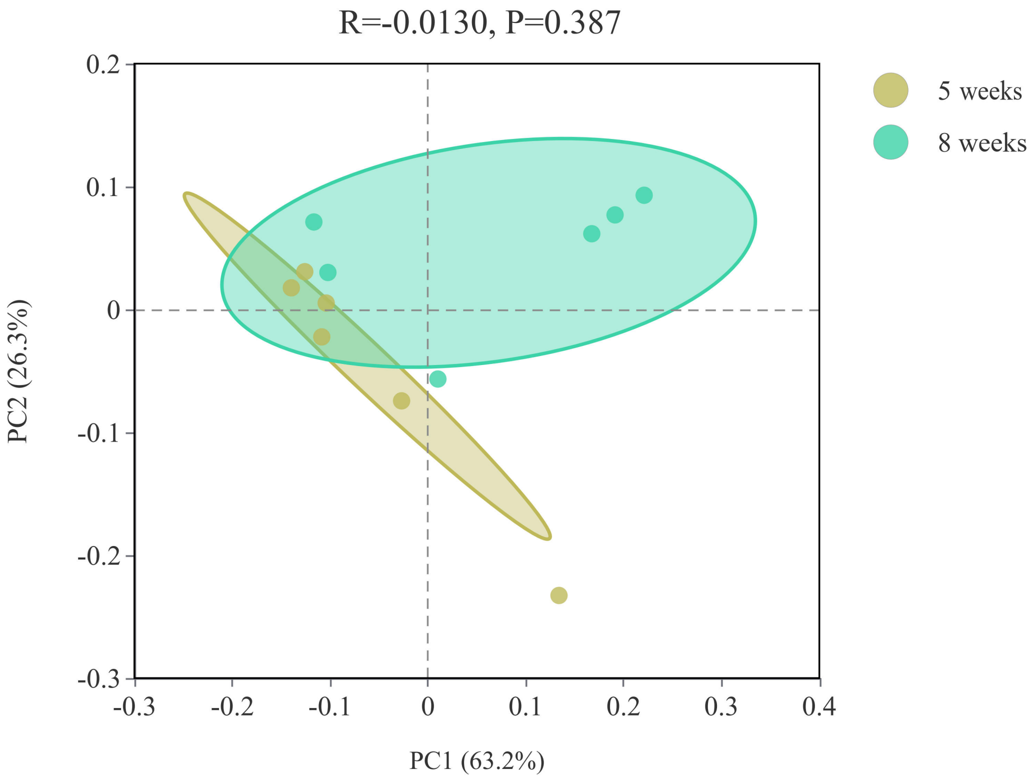

Supplementary figure 3. PCA analysis of gut microbiota from 5-weeks-old and 8-weeks-old mice. P value was calculated using Student's t test. N = 5 per group.

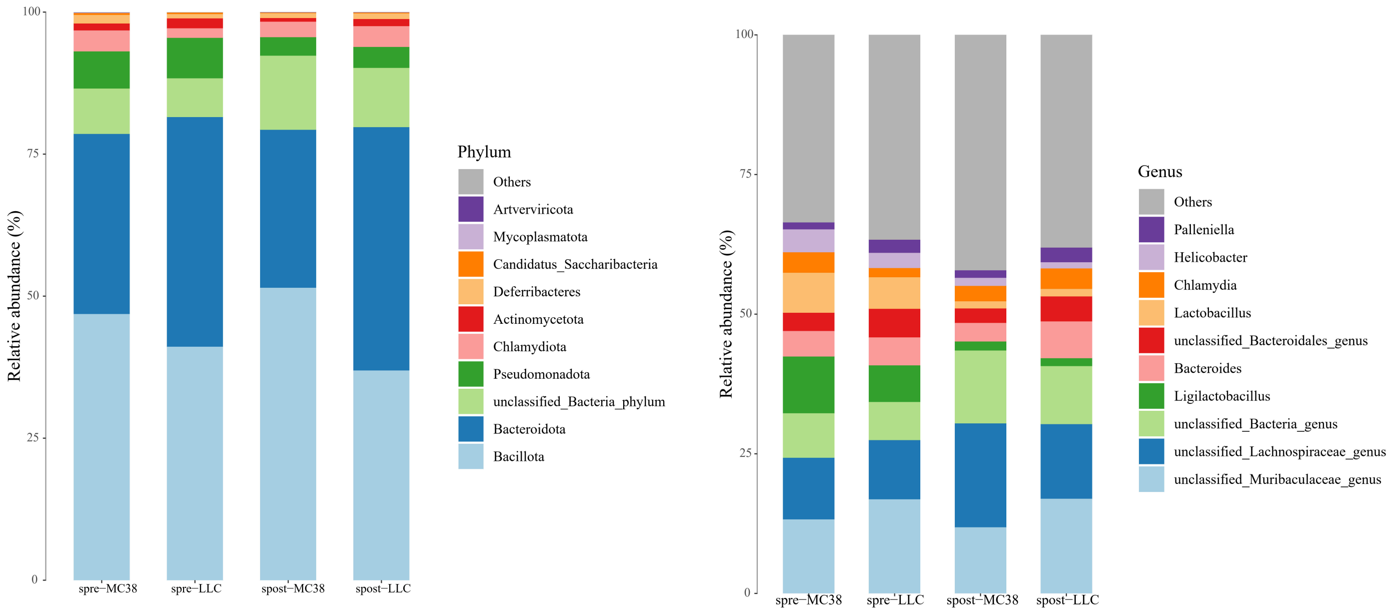

Supplementary figure 4. The gut microbiota composition (top 10) at phylum (left) and genus (right) level of subcutaneous tumor.

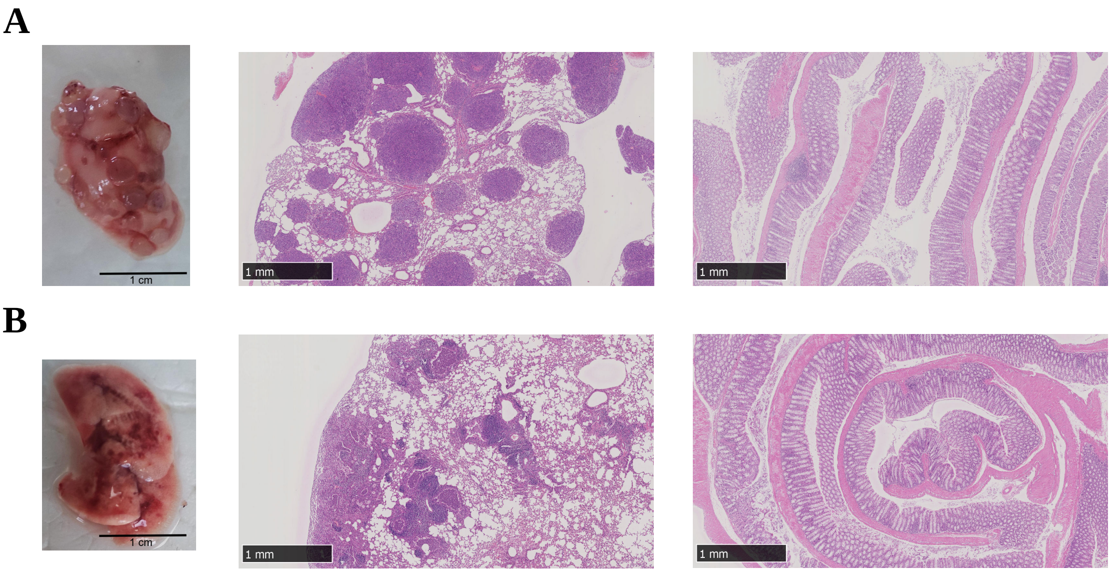

Supplementary figure 5. Metastatic tumor. The photograph (left, scale bar = 1 cm) and HE staining of lung (middle, scale bar = 1 mm) and colorectum (right, scale bar = 1 mm) of MC38 (A) and LLC (B) metastatic tumor.

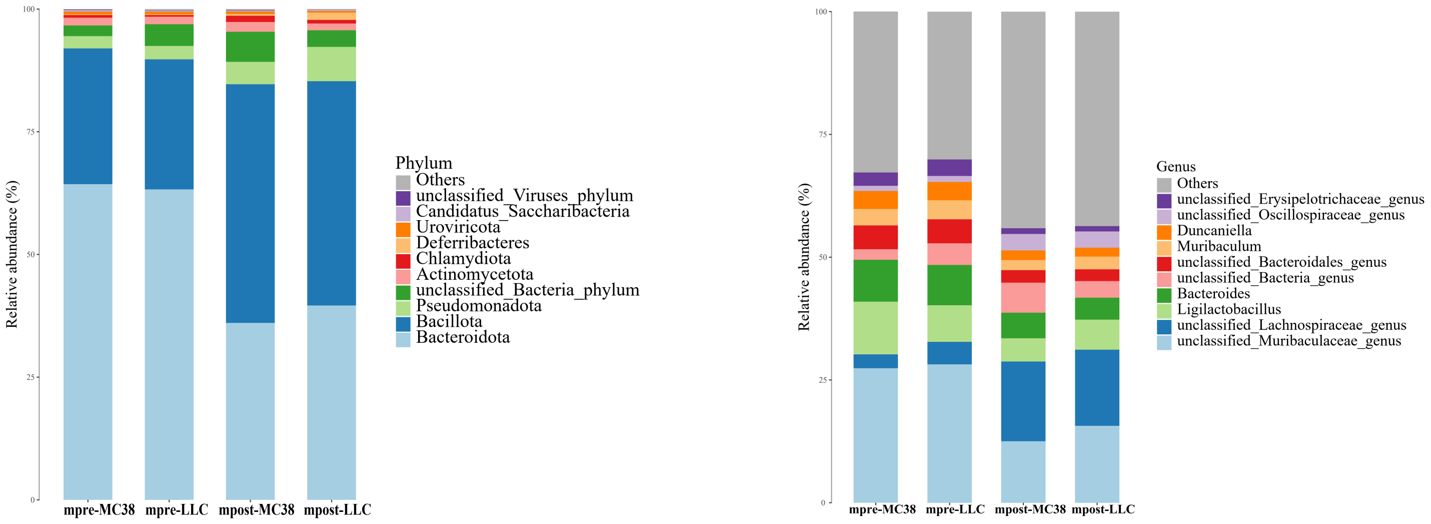

Supplementary figure 6. The gut microbiota composition (top 10) at phylum (left) and genus (right) level of metastatic tumor.

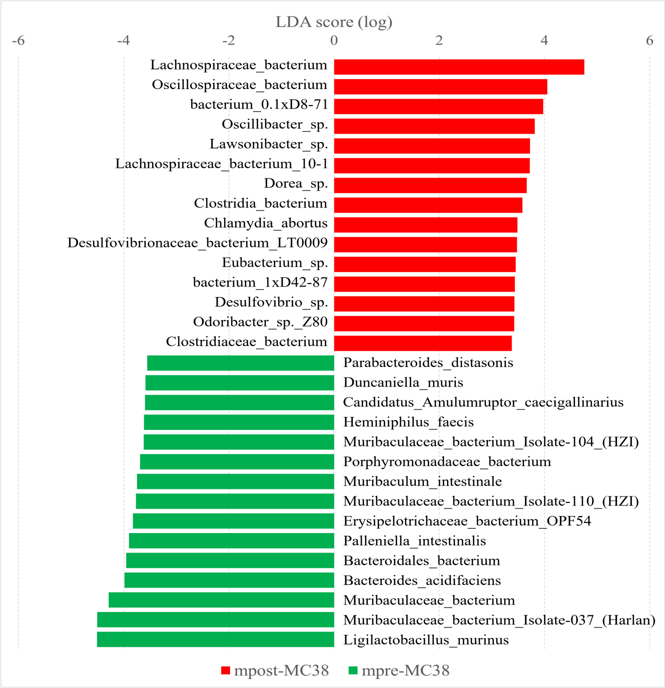

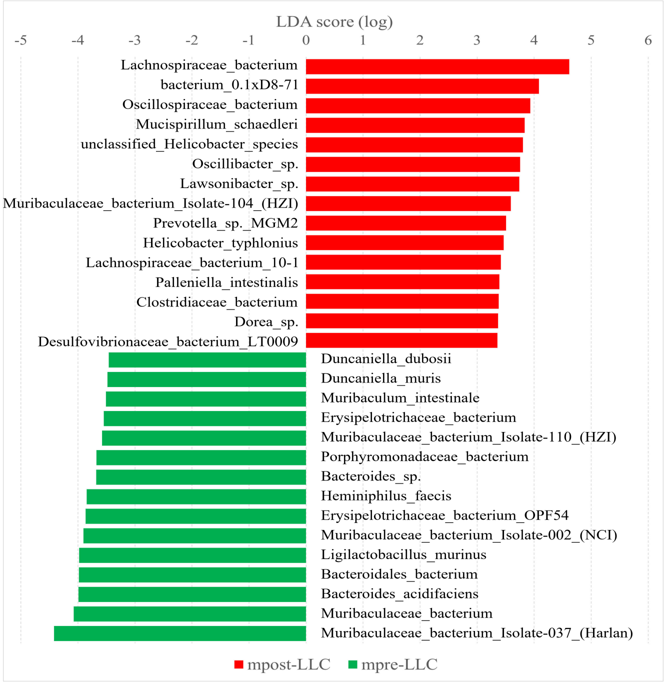

Supplementary figure 7. The top 15 histograms of LDA coupled with effective size between mpost-MC38 and mpre-MC38 (left), and between mpost-LLC and mpre- LLC (right).

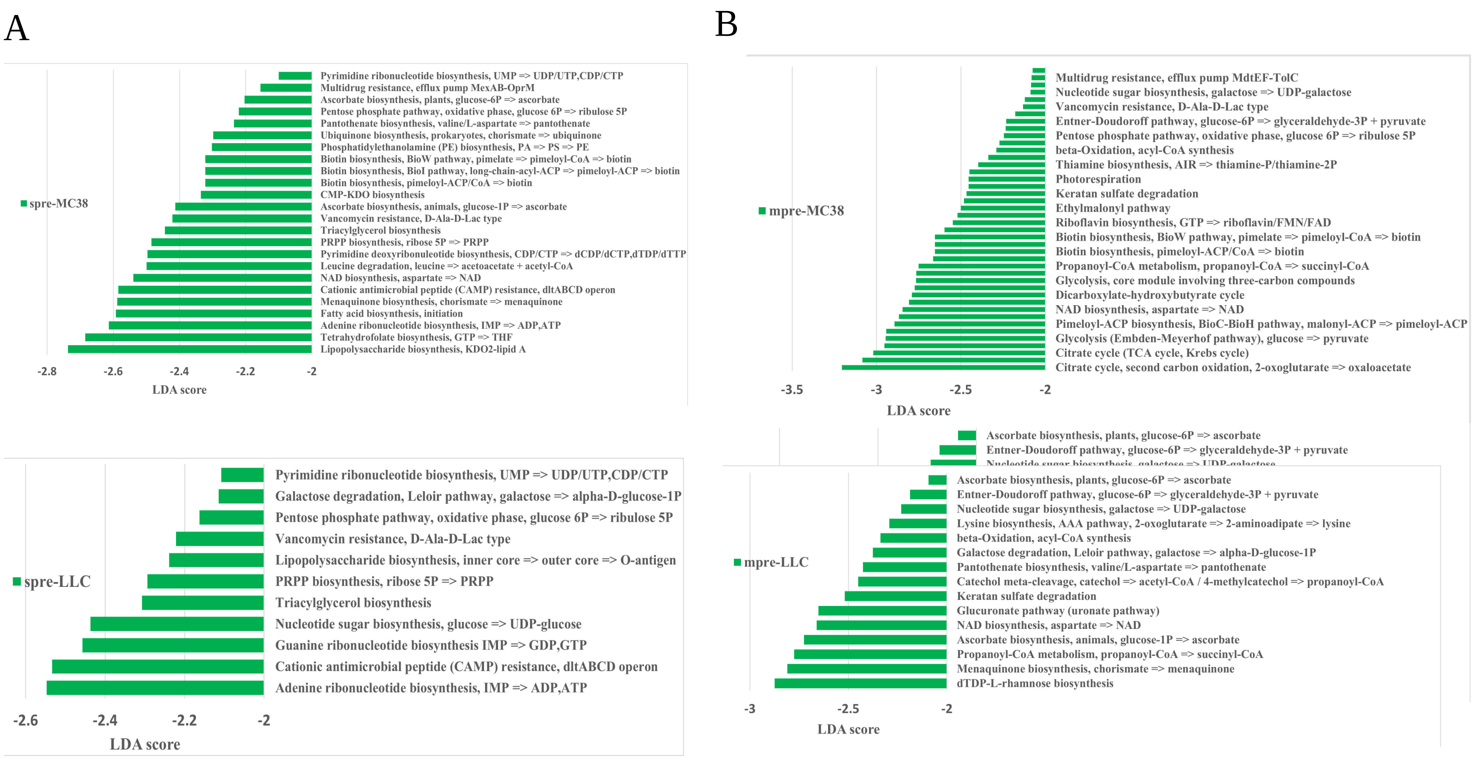

Supplementary figure 8. Enriched KEGG pathway modules of metabolic function of gut microbiota in pre-inoculation groups. (A) Enriched modules in gut microbiota in spre-MC38 and spre-LLC groups compared with their respective post-inoculation groups. (B) Enriched modules in gut microbiota in mpre-MC38 and mpre -LLC groups compared with their respective post-inoculation groups.

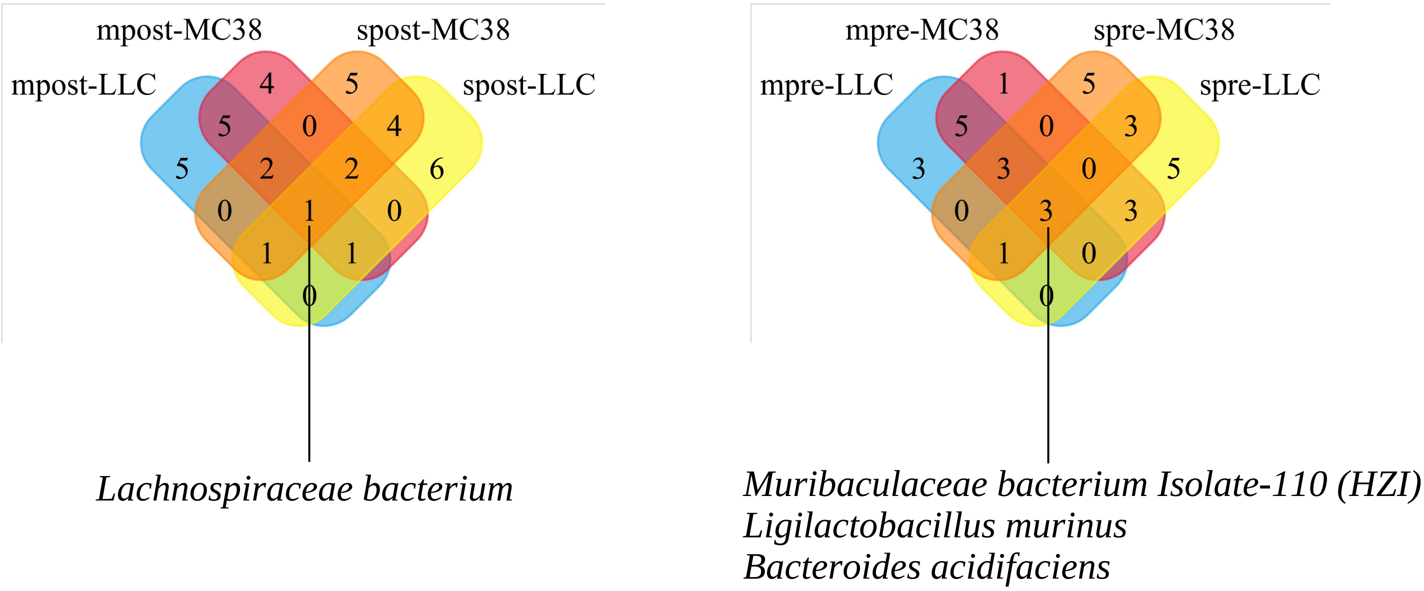

Supplementary figure 9 Veen diagram of top 15 most abundance species among spost-MC38, spost-LLC, mpost-MC38 and mpost-LLC groups (left), as well as among spre-MC38, spre-LLC, mpre-MC38 and mpre-LLC groups (right), compared with their respective post-inoculation groups.

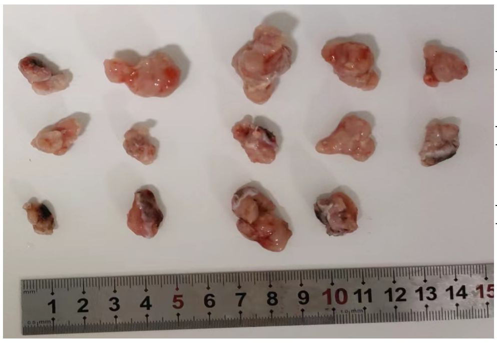

FMT-PBS-MC38

FMT-spre-MC38

FMT-spost-MC38

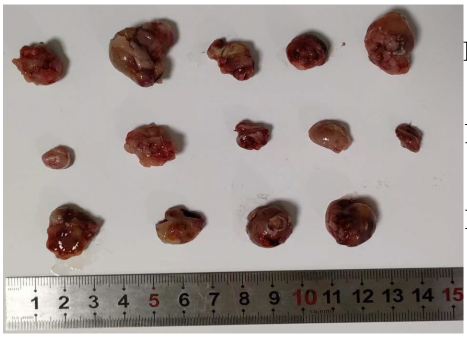

FMT-PBS-LLC

FMT-spre-LLC

FMT-spost-LLC

Supplementary figure 10. Image of tumor from different groups inoculated MC38 (left) or LLC (right) in FMT. One mouse in the FMT-spost-MC38 group and one in the FMT-spost-LLC group died during the gavage process.
